# Conbercept for patients with age-related macular degeneration: a systematic review

**DOI:** 10.1186/s12886-018-0807-1

**Published:** 2018-06-15

**Authors:** Jiaxing Zhang, Yi Liang, Juan Xie, Dong Li, Qian Hu, Xiaosi Li, Wenyi Zheng, Rui He

**Affiliations:** 1 Department of Pharmacy, Guizhou provincial people’s hospital, No.83 Zhongshandong Road, Nanming District, Guiyang, Guizhou Province China; 2 Health Outcomes and Pharmacy Practice, College of Pharmacy, the University of Texas at Austin, Austin, Texas USA; 3 Department of Ophthalmology, Guizhou provincial people’s hospital, Guiyang, Guizhou Province China; 4 Department of Pharmacy, Hospital of Chengdu Office of People’s Government of Tibetan Autonomous Region, No.20 Ximianqiaoheng Street, Wuhou District, Chengdu, Sichuan Province China; 5 Department of Laboratory Medicine, Experimental Cancer Medicine, Clinical Research Center, Karolinska Institute, 14186 Huddinge, Stockholm Sweden

**Keywords:** Wet age-related macular degeneration, Vascular endothelial growth factor (VEGF) inhibitor, Conbercept, Ranibizumab, Systematic review

## Abstract

**Background:**

Conbercept is a novel vascular endothelial growth factor (VEGF) inhibitor for the treatment of wet age-related macular degeneration (AMD). This systematic review aims to assess the efficacy and safety of conbercept in the treatment of wet AMD.

**Methods:**

PubMed, Embase, Cochrane Library, China National Knowledge Infrastructure, VIP database, and Wanfang database were searched from their earliest records to June 2017. We included randomized controlled trials (RCTs) evaluating the efficacy and safety of conbercept in wet AMD patients. Outcomes included the mean changes from baseline in best-corrected visual acuity (BCVA) score (primary outcome), central retinal thickness (CRT), plasma level of vascular endothelial growth factor (VEGF) over time, and the incidence of adverse events (AEs).

**Results:**

Eighteen RCTs (1285 participants) were included in this systematic review. Conbercept might improve BCVA compared to triamcinolone acetonide [*MD* = 0.11, 95% *CI* (0.08, 0.15)], and reduce CRT compared to the other four therapies (conservative treatment, ranibizumab, transpupillary thermotherapy, and triamcinolone acetonide). The incidence of AEs in patients receiving conbercept was significantly lower than those receiving triamcinolone acetonide [*RR* = 0.25, 95% *CI* (0.09–0.72)], but was similar to the other therapies. Conbercept seemed to be more effective than ranibizumab in lowering the plasma level of VEGF [*MD* = − 15.86, 95% *CI* (− 23.17, − 8.55)].

**Conclusions:**

Current evidence shows that conbercept is a promising option for the treatment of wet AMD. Nevertheless, further studies are required to compare the efficacy, long-term safety and cost-effectiveness between conbercept and other anti-VEGF agents in different populations.

**Electronic supplementary material:**

The online version of this article (10.1186/s12886-018-0807-1) contains supplementary material, which is available to authorized users.

## Background

Age-related macular degeneration (AMD) is a progressive chronic disease of the central retina (the macula) and will result primarily in loss of central vision. It has become the leading cause of adult blindness in industrialized countries [1]. The incidence is expected to at least double by 2020 [2]. The Global Burden of Disease Study 2010 reported an exponential increase of 160% in vision-related years lived with disability due to AMD, highlighting the overwhelming burden to society [3]. A systematic review also revealed that, in 2010, 2.1 million people were blind and 6.0million people were visually impaired due to macular diseases, excluding those caused by diabetic maculopathy [4]. Its prevalence increased from 1990 to 2010 with the highest increase in high-income regions and among the older population (≥50 years of age). The prevalence is comparable between Asians and whites [5], but lower in blacks [6]. However, Asians are more likely to have less-common AMD variants (polypoidal choroidal vasculopathy, PCV) [7, 8].

Clinically, AMD is classified into dry (atrophic) or wet (neovascular or exudative, which accounts for more than 80% of cases with severe visual loss or legal blindness [9]). Established therapies for wet AMD include intravitreous injection of a vascular endothelial growth factor (VEGF) inhibitor, possibly thermal laser photocoagulation, photodynamic therapy (PDT), and supplementation with zinc and antioxidant vitamins. PDT is an alternative for patients who cannot be treated with an intravitreal VEGF inhibitor and for patients with chronic exudative lesions who have preserved vision in one eye and are unlikely to achieve reading vision in the second eye. Transpupillary thermotherapy and triamcinolone acetonide are also used for wet AMD, but recurrence rates of both therapies are relatively high. According to the guidelines from American Academy of Ophthalmology (AAO) and the European Society of Retina Specialists (EURETINA), VEGF inhibitors (e.g., aflibercept, bevacizumab, and ranibizumab) are most effective to manage neovascular AMD and are considered first-line of treatment [2, 10].

VEGF (a potent mitogen and vascular permeability factor) plays a pivotal role in neovascularization by increasing vascular permeability, enhancing the inflammatory response and inducing angiogenesis [11]. Inhibiting VEGF can limit the progression of wet AMD and stabilize, or reverse visual loss [12]. Between 2004 and 2006, three anti-VEGF drugs (Pegaptanib, ranibizumab, and bevacizumab) with different sites of action, formulations, binding affinities, and biologic activities were introduced for the treatment of wet AMD. In November 2011, aflibercept, which binds to all VEGF-A and VEGF-B isoforms as well as to the highly related placental growth factor (PIGF) was approved by the US Food and Drug Adminstration. Similar to aflibercept, conbercept (KH902), a recombinant fusion protein with high affinity to all VEGF isoforms and PIGF [13], was developed and approved in China for the treatment of wet AMD in December 2013. Several randomized trials investigating the use of conbercept concluded that it was effective and safe in the treatment of wet AMD. Nevertheless, evidence has not been systematically assessed. To understand and interpret available evidence, we conducted a systematic review to evaluate the efficacy and safety of conbercept in patients with wet AMD.

## Methods

We followed the standard set by Preferred Reporting Items for Systematic reviews and Meta-Analyses (PRISMA) in this systematic review (Additional file 1: Table S1). The study was registered in PROSPERO International Prospective Register of Systematic Review (PROSPERO 2017: CRD42017071144).

### Literature searching

Pubmed, Embase, and Cochrane Central Register of Controlled Trials (CENTRAL) published in Cochran Library were searched using the search strategies detailed in Additional file 1: Table S2, from their earliest records to June 2017. Clinicaltrials.gov was searched with the terms “age-related macular degeneration” and “conbercept”. The China National Knowledge Infrastructure(CNKI), VIP database, and Wanfang database were also searched with Chinese terms.

### Eligibility criteria

All included studies met the following criteria: (1) randomized controlled studies (RCTs); (2) participants with wet age-related macular degeneration aged more than 50 years old; (3) the intervention was conbercept irrespective of dosage and schedule; (4) the comparisons included conservative treatment, ranibizumab, transpupillary thermotherapy, and triamcinolone acetonide; (5) studies included at least one of the following outcomes: the primary outcome was the mean change in best-corrected visual acuity (BCVA, which was measured by the logarithmic visual acuity chart) from baseline to the third month after the first treatment; secondary outcomes included the mean change in central retinal thickness (CRT, which was measured by optical coherence tomography) from baseline to the last visit, the mean change in plasma level of VEGF from baseline to the last visit, and the incidence of adverse events (AEs); (6) publication written in English or Chinese. We excluded the patients with glaucoma, cataracts, or retinopathy caused by diabetes or hypertension and studies without available raw data.

### Study selection and data extraction

Two investigators independently screened the titles and abstracts of the articles identified by literature searching (Additional file 1: Table S2 shows the searching strategy), and assessed the studies using predetermined inclusion criteria. The full texts of all potentially relevant articles were retrieved for detailed review. Any disagreement in the process of selection was resolved by discussion. Two authors independently extracted following data from included articles: (1) authors; (2) year of publication; (3) country or region where the study was conducted; (4) study design and use of control; (5) number of participants randomized into each group; (6) gender, age, and disease duration of participants; (7) treatment regimens (dose and schedule); (8) outcomes of each study and their definitions; (9) numerical data for outcomes assessment; (10) sources of funding.

### Risk of Bias assessment

Two authors independently assessed the bias risk of each included study using the checklist developed by Cochrane Collaboration [14, 15]. Any disagreements about the risk of bias was resolved by discussion. The items included random sequence generation, allocation concealment, blinding, incomplete outcome data, selective outcome reporting, and other bias. We categorized the judgments as low, high or unclear risk of bias and created plots of bias risk assessment in Review Manager Software (RevMan 5.3).

### Statistical synthesis

We calculated a kappa statistic for measuring the agreement level between two authors regarding to the decisions made on study selection. The value of kappa (*K*) 0.40–0.59 was considered fair agreement, 0.60–0.74 as good and 0.75 or more as excellent [16].

If more than one study reported the same outcome, a pairwise meta-analysis was conducted. We analyzed RCTs using risk ratios (*RRs*) for the incidence of AEs and mean differences (*MDs*) for BCVA, CRT, and VEGF level, with corresponding 95% confidence intervals (*CIs*) to compare differences between conbercept and control groups. We pooled *RRs* with the Mantel-Haenszel method, and *MDs* with the inverse variance method using RevMan 5.3, respectively. Statistical heterogeneity among studies was examined by the Chi-square test and quantified by the *I*^*2*^ statistic [14]. We applied a fixed-effects model to synthesize data when heterogeneity was not significant (*P>*0.1 and *I*^*2*^<50%). When heterogeneity was significant (*P* ≤ 0.1 and *I*^*2*^ ≥ 50%) and could not be explained by subgroup analyses or in terms of clinical or methodological features of the trials, a random-effects model was used. We explored sources of heterogeneity based on the following subgroup analyses: type of control groups (e.g. conservative treatment, triamcinolone acetonide, transpupillary thermotherapy, or ranibizumab). We carried out sensitivity analyses by using alternative pooling methods (Peto vs. Mantel-Haenszel method), and statistical models regarding to heterogeneity (random-effects vs. fixed-effect).

## Results

### Search results and characteristics of included studies

**Fig. 1:**
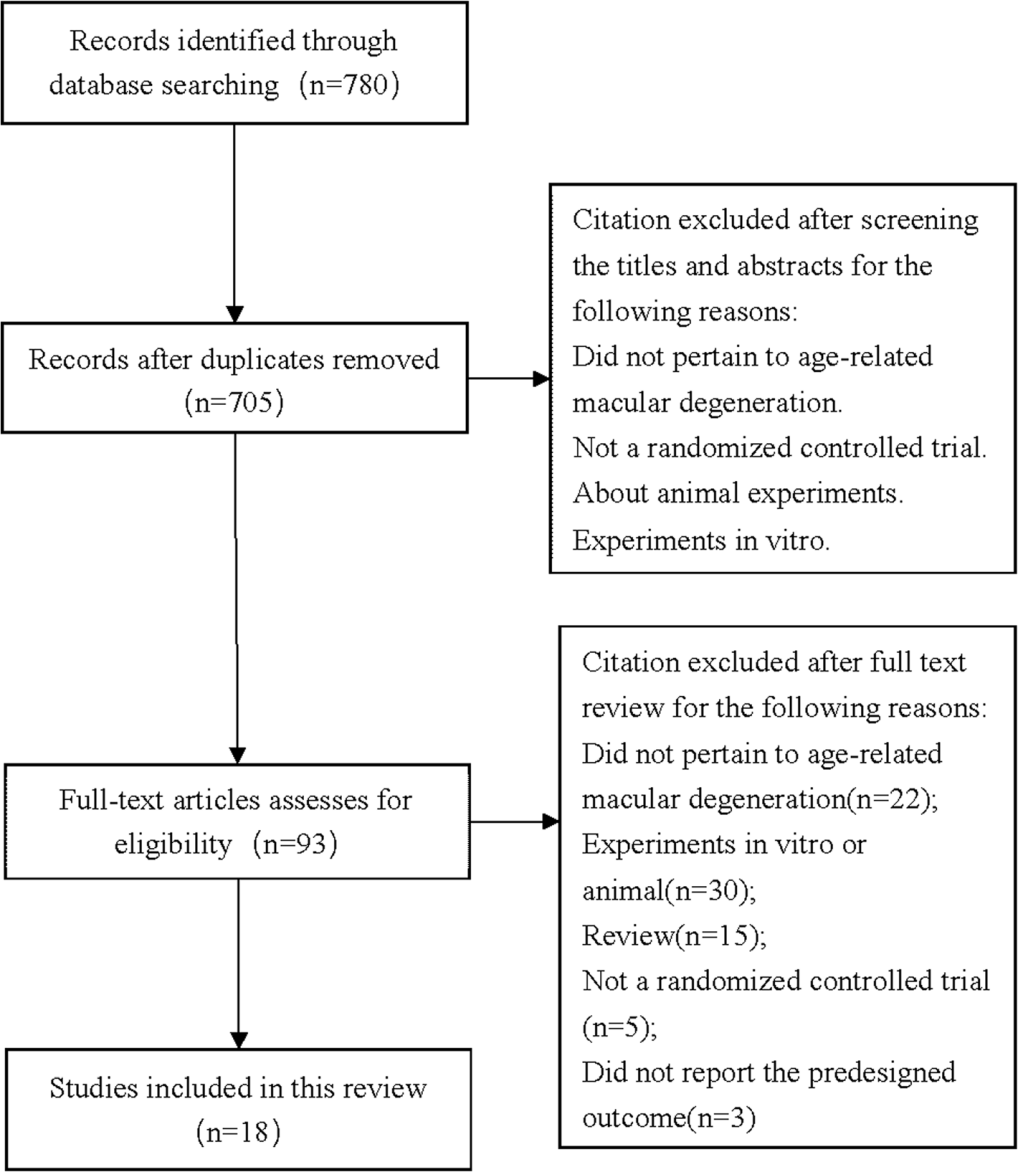
Flow diagram of study selection process for this systematic review

A total of 780 citations were obtained from the literature search and the selection process is shown in Fig. 1. Eighteen RCTs (1285 participants) [17–34] were included in this systematic review. Agreement on study selection between two reviewers was excellent (*K* = 0.83). All the RCTs were single-center studies conducted in China. As shown in Table 1, the comparisons were conservative treatment (3 RCTs [17–19], 232 participants), ranibizumab (6 RCTs [20–25], 395 participants), transpupillary thermotherapy (4 RCTs [26–29], 326 participants), and triamcinolone acetonide (5 RCTs [30–34], 332 participants). The follow-up time ranged from 1 to 12 months after the first treatment, except one study [18] without reporting this information.

**Table 1:** Characteristic of included studies

Study ID	Study design	Participants	*N*	Intervention group (*N*)	Comparison group (*N*)	Outcomes	Follow-up time after the first treatment (months)
Li YY, 2017 [17]	RCT	wet AMD	54	Conbercept (27)	Conservative treatment (27)	②④	6
Mei HY, 2017 [18]	RCT	wet AMD	66	Conbercept (33)	Conservative treatment (33)	②	NA
Song W, 2016 [19]	RCT	wet AMD	112	Conbercept (56)	Conservative treatment (56)	②④	6
Liu ZN, 2016 [20]	RCT	wet AMD	40	Conbercept (20)	Ranibizumab (20)	②	12
Zhang HX, 2016 [21]	RCT	wet AMD	50	Conbercept (25)	Ranibizumab (25)	②③④	1
Liu R, 2015 [22]	RCT	wet AMD	60	Conbercept (30)	Ranibizumab (30)	②③	3
Wang NF, 2017 [23]	RCT	wet AMD	76	Conbercept (38)	Ranibizumab (38)	①②	3
Lyu P, 2016 [24]	RCT	wet AMD	84	Conbercept (42)	Ranibizumab (42)	①②④	3
Zheng MW, 2017 [25]	RCT	wet AMD	85	Conbercept (42)	Ranibizumab (43)	①②	3
Wang XX, 2015 [26]	RCT	wet AMD	60	Conbercept (30)	Transpupillary thermotherapy (30)	②	6
Qin MM, 2016 [27]	RCT	wet AMD	82	Conbercept (41)	Transpupillary thermotherapy (41)	②	3
Li L, 2017 [28]	RCT	wet AMD	86	Conbercept (43)	Transpupillary thermotherapy (43)	②④	6
Zhang X, 2015 [29]	RCT	wet AMD	98	Conbercept (49)	Transpupillary thermotherapy (49)	②	6
Zhu Y, 2017 [30]	RCT	wet AMD	50	Conbercept (25)	Triamcinolone acetonide (25)	①②④	3
He XT, 2015 [31]	RCT	wet AMD	60	Conbercept (30)	Triamcinolone acetonide (30)	①②④	3
Han X, 2017 [32]	RCT	wet AMD	70	Conbercept (35)	Triamcinolone acetonide (35)	①②④	3
Pan XL, 2017 [33]	RCT	wet AMD	76	Conbercept (38, 42eyes)	Triamcinolone acetonide (38, 38eyes)	②	12
Yue JL, 2017 [34]	RCT	wet AMD	76	Conbercept (38)	Triamcinolone acetonide (38)	②	12

*RCT* randomized controlled trials, *AMD* age-related macular degeneration, *N* number of participants, *NA* not available; ① the mean change in best-corrected visual acuity from baseline to the third month after the first treatment; ② the mean change in central retinal thickness from baseline to the last visit; ③ the mean change in plasma level of VEGF from baseline to the last visit; ④ adverse events

As shown in Table 2, all participants were aged 51–87 years old with disease duration of 7 days to 10 years (10 studies [17, 19, 23–26, 28–31] did not report the disease duration). The reported dose of conbercept ranged from 0.5 to 1.5 mg, except 2 studies [21, 30] which failed to report dosing information.

**Table 2:** Therapeutic regimen and characteristic of included participants

Study ID	Therapeutic regimen of intervention group	Therapeutic regimen of comparison group	Gender(male/female): intervention group vs comparison group	Age(years): intervention group vs comparison group	Course of disease: intervention group vs comparison group
Li YY, 2017 [17]	Conbercept, 0.5 mg, intravitreal injection, once a month, continuous treatment for 3 months.	Conservative treatment	16/11 vs 14/13	76.8 ± 12.6(58–85) vs 77.6 ± 11.9(59–83)	NA
Mei HY, 2017 [18]	Conbercept, 0.5 mg, intravitreal injection, once a month, continuous treatment for 3 months.	Conservative treatment	17/16 vs 16/17	69.4 ± 4.3(63–72) vs 69.5 ± 4.1(63–71)	3.1 ± 0.5y(2-4y) vs 3.2 ± 0.6y(2-5y)
Song W, 2016 [19]	Conbercept, 0.5 mg, intravitreal injection, once a month, continuous treatment for 3 months.	Conservative treatment	30/26 vs 29/27	62.36 ± 6.56(53–81) vs 63.29 ± 6.45(54–82)	NA
Liu ZN, 2016 [20]	Conbercept, 0.5 mg, intravitreal injection, once a month, continuous treatment for 3 months.	Ranibizumab, 0.5 mg, intravitreal injection, once a month, continuous treatment for 3 months.	11/9 vs 12/8	65.41 ± 6.37(57–86) vs 65.36 ± 6.74(56–85)	3.81 ± 1.10y(6 m-10y) vs 3.88 ± 1.20y(7 m-11y)
Zhang HX, 2016 [21]	Conbercept, NA	Ranibizumab, NA	12/13 vs 13/12	68.79 ± 7.21(54–81) vs 69.03 ± 7.01(53–82)	6.13 ± 4.27 m(7d-14 m) vs 6.25 ± 4.45 m(3d-15 m)
Liu R, 2015 [22]	Conbercept, 1.5 mg, intravitreal injection, once a month, continuous treatment for 3 months.	Ranibizumab, 0.5 mg, intravitreal injection, once a month, continuous treatment for 3 months.	NA	65.5 ± 8.1(53–83) vs 65.2 ± 8.3(54–81)	18.8 ± 4.1 m(8-31 m) vs 19.0 ± 4.2 m (9-32 m)
Wang NF, 2017 [23]	Conbercept, 0.5 mg, intravitreal injection	Ranibizumab, 0.5 mg, intravitreal injection	19/19 vs 20/18	62.89 ± 5.46(51–75) vs 62.85 ± 5.41(52–74)	NA
Lyu P, 2016 [24]	Conbercept, 0.5 mg, intravitreal injection, once a month, continuous treatment for 3 months.	Ranibizumab, 0.5 mg, intravitreal injection, once a month, continuous treatment for 3 months.	22/20 vs 21/21	62.7 ± 5.3(51–73) vs 62.1 ± 4.9(49–70)	NA
Zheng MW, 2017 [25]	Conbercept, 0.5 mg, intravitreal injection, once a month, continuous treatment for 3 months.	Ranibizumab, 0.5 mg, intravitreal injection, once a month, continuous treatment for 3 months.	25/17 vs 26/17	65.34 ± 2.29(61–72) vs 65.13 ± 2.27(61–74)	NA
Wang XX, 2015 [26]	Conbercept, 0.5 mg, intravitreal injection, once a month, continuous treatment for 3 months.	Transpupillary thermotherapy, course of treatment: 3 months	17/13 vs 16/14	70.4 ± 8.4 vs 69.7 ± 8.1	NA
Qin MM, 2016 [27]	Conbercept, 1.0 mg, intravitreal injection, once a month, continuous treatment for 3 months.	Transpupillary thermotherapy, course of treatment: 3 months	20/21 vs 22/19	65.8 ± 7.5(54–81) vs 64.9 ± 7.7(51–79)	5.7 ± 4.4y(9 m-25y) vs 4.7 ± 3.2y(11 m-21y)
Li L, 2017 [28]	Conbercept, 0.5 mg, intravitreal injection, once a month, continuous treatment for 3 months.	Transpupillary thermotherapy, course of treatment: 3 months	NA	62.28 ± 3.21(52–82) vs 63.12 ± 4.36(53–83)	NA
Zhang X, 2015 [29]	Conbercept, 0.5 mg, intravitreal injection, once a month, continuous treatment for 3 months.	Transpupillary thermotherapy, course of treatment: 3 months	NA	64.19 ± 4.31(54–85) vs 65.43 ± 5.76(55–86)	NA
Zhu Y, 2017 [30]	Conbercept, NA	Triamcinolone acetonide, 0.1 ml, intravitreal injection.	NA	NA	NA
He XT, 2015 [31]	Conbercept, 1.0 mg, intravitreal injection.	Triamcinolone acetonide, 0.1 ml, intravitreal injection.	14/16 vs 13/17	61.52 ± 6.8(57–72) vs 61.30 ± 6.2(55–70)	NA
Han X, 2017 [32]	Conbercept, 0.5 mg, intravitreal injection, once a month, continuous treatment for 3 months.	Triamcinolone acetonide, 0.1 ml, intravitreal injection, once a month, continuous treatment for 3 months.	16/19 vs 14/21	67.54 ± 4.45(55–83) vs 68.12 ± 4.66(54–87)	3.75 ± 0.64y(0.5-10y) vs 3.94 ± 0.71y(0.5-12y)
Pan XL, 2017 [33]	Conbercept, 1.0 mg, intravitreal injection	Triamcinolone acetonide, 0.1 ml, intravitreal injection.	20/18 vs 22/16	71.62 ± 5.27(63–85) vs 71.59 ± 5.11(62–83)	15.83 m ± 5.25 m(5-25 m) vs 15.65 m ± 5.34 m(7-29 m)
Yue JL, 2017 [34]	Conbercept, 0.5 mg, intravitreal injection	Triamcinolone acetonide, 0.1 ml, intravitreal injection.	21/17 vs 22/16	69.78 ± 7.52(61–82) vs 69.27 ± 8.36(60–81)	45.62 ± 14.79 m(5-126 m) vs 46.15 ± 14.34 m(7-123 m)

*NA* not available, *d* day, *m* month, *y* year

### Risk of Bias

**Fig. 2:**
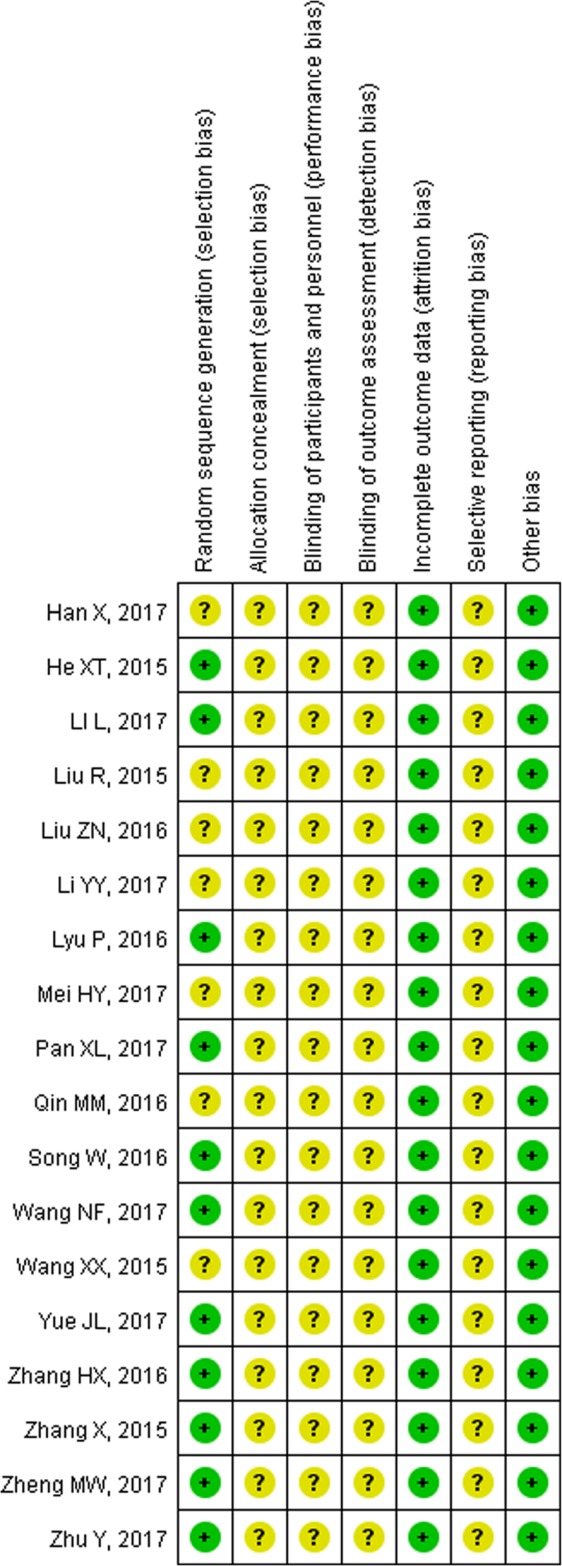
Risk of bias summary

As shown in Fig. 2, the random sequences of 11 studies [19, 21, 23, 24, 28–31, 33, 34] were generated by a random number table or simulation, while all the studies failed to describe the method of allocation concealment. Therefore, the risk of selection bias related to allocation concealment was unable to be assessed. The risk of performance bias of all studies was uncertain, as the blinding method was not reported. All studies had low risk of attrition bias, as there was no loss to follow-up. No studies contained information related to registration information nor had protocols available, so it was unknown whether all the pre-designed outcomes in these studies had been reported. Since none of the studies were reported being supported by pharmaceutical industry funding, the bias caused by conflict of interest was low. Due to the limited number of the included studies for the same outcome, publication bias investigation was not performed.

### Best-corrected visual acuity (BCVA)

**Fig. 3:**
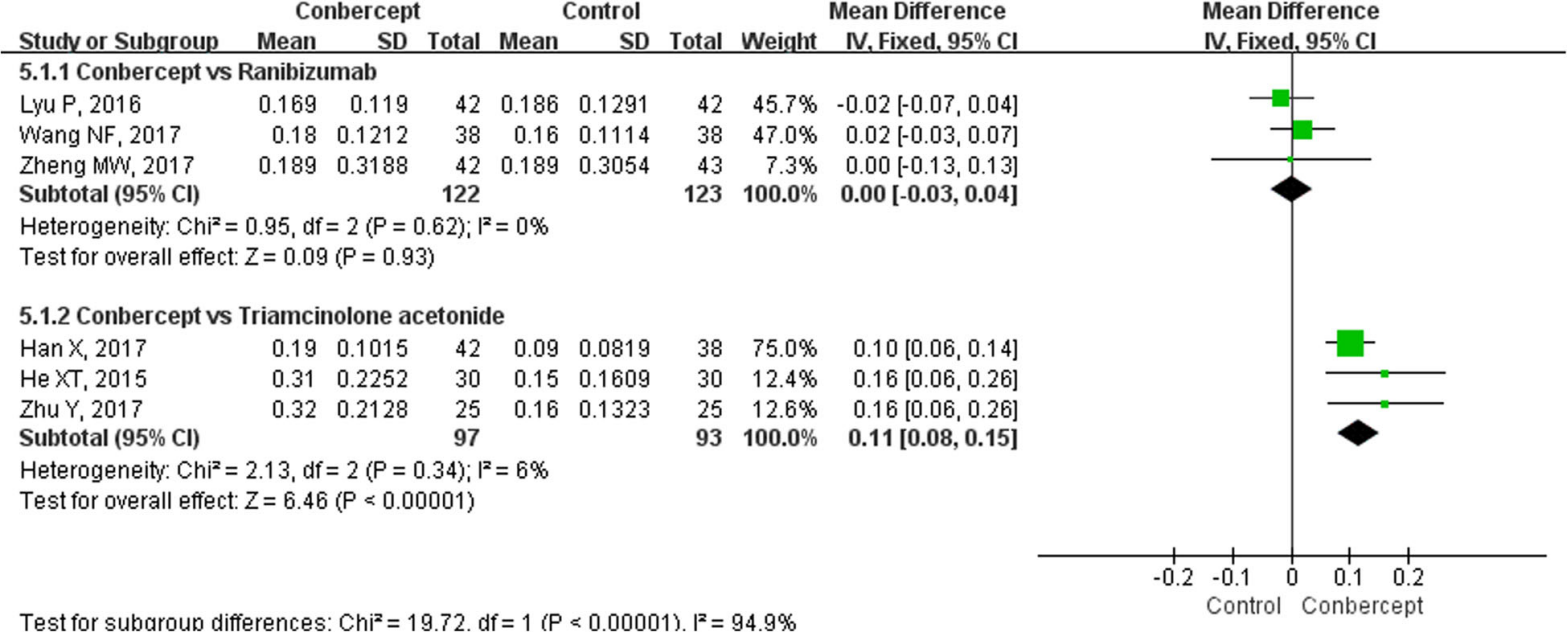
The forest plot of best-corrected visual acuity

The mean change in BCVA from baseline to the third month after the first treatment was reported in 6 studies [23–25, 30–32] (435 participants). Subgroup analyses were performed and stratified by control group selection (Fig. 3). The heterogeneity of each subgroup was not statistically significant (*I*^*2*^<50%, *P*>0.1), so the *MD*s of the mean changes of BCVA were pooled with a fixed-effects model. The difference between the pooled results of the two subgroups was significant (*P* < 0.00001). Compared to triamcinolone acetonide, conbercept significantly improved the BCVA in the third month after the first treatment [*MD* = 0.11, 95%*CI* (0.08, 0.15)]. While the mean change in BCVA from baseline in conbercept group was similar to that in ranibizumab group [*MD* = 0.00, 95% *CI* (− 0.03, 0.04)].

### Central retinal thickness (CRT)

**Fig. 4:**
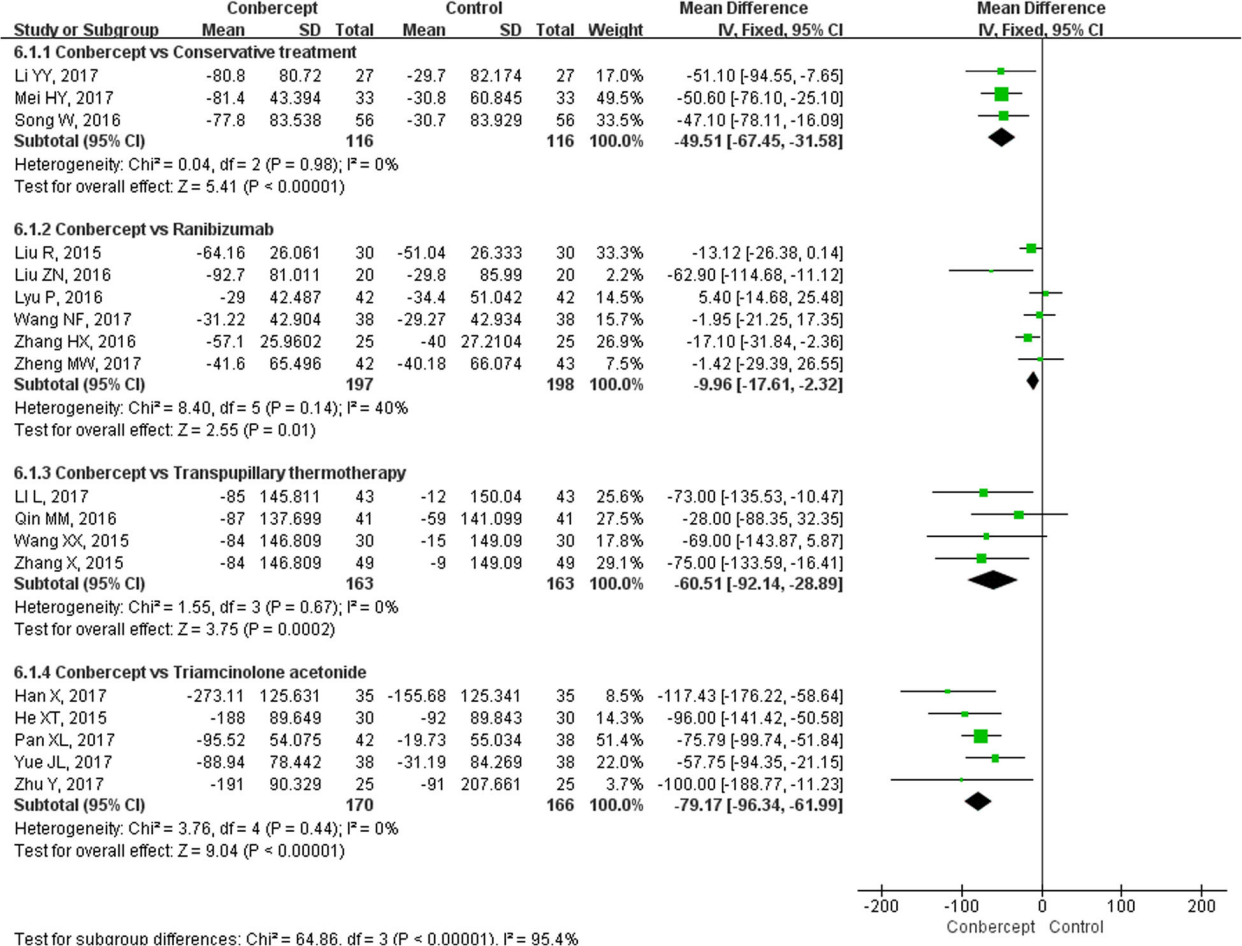
The forest plot of central retinal thickness

The mean change in CRT from baseline to the last visit was reported in all 18 studies [17–34] (1285 participants). Subgroup analyses were performed and stratified by control group selection (Fig. 4). The *MD*s of the mean changes of CRT were pooled with a fixed-effects model because the heterogeneity of each subgroup was not statistically significant (*I*^*2*^<50%, *P*>0.1). There were significant differences among the pooled results of the four subgroups (*P* < 0.00001). Compared with the other four therapies (conservative treatment, ranibizumab, transpupillary thermotherapy, triamcinolone acetonide), conbercept significantly reduced the CRT at the last visit [*MD* = − 49.51, 95% *CI* (− 67.45, − 31.58); *MD* = − 9.96, 95% *CI* (− 17.61, − 2.32); *MD* = − 60.51, 95% *CI* (− 92.14, − 28.89); *MD* = − 79.17, 95% *CI* (− 96.34, − 61.99), respectively].

### Plasma level of VEGF(ng/L)

**Fig. 5:**
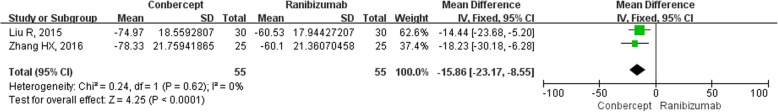
The forest plot of plasma level of vascular endothelial growth factor (VEGF)

Two studies [21, 22] (110 participants) reported the plasma level of VEGF. According to Fig. 5, the heterogeneity was not statistically significant (*I*^*2*^ = 0%, *P* = 0.62). The pooled results with a fixed-effects model indicated that conbercept significantly lowered the plasma level of VEGF [*MD* = − 15.86, 95% *CI* (− 23.17, − 8.55)] compared to ranibizumab.

### Adverse events (AEs)

**Fig. 6:**
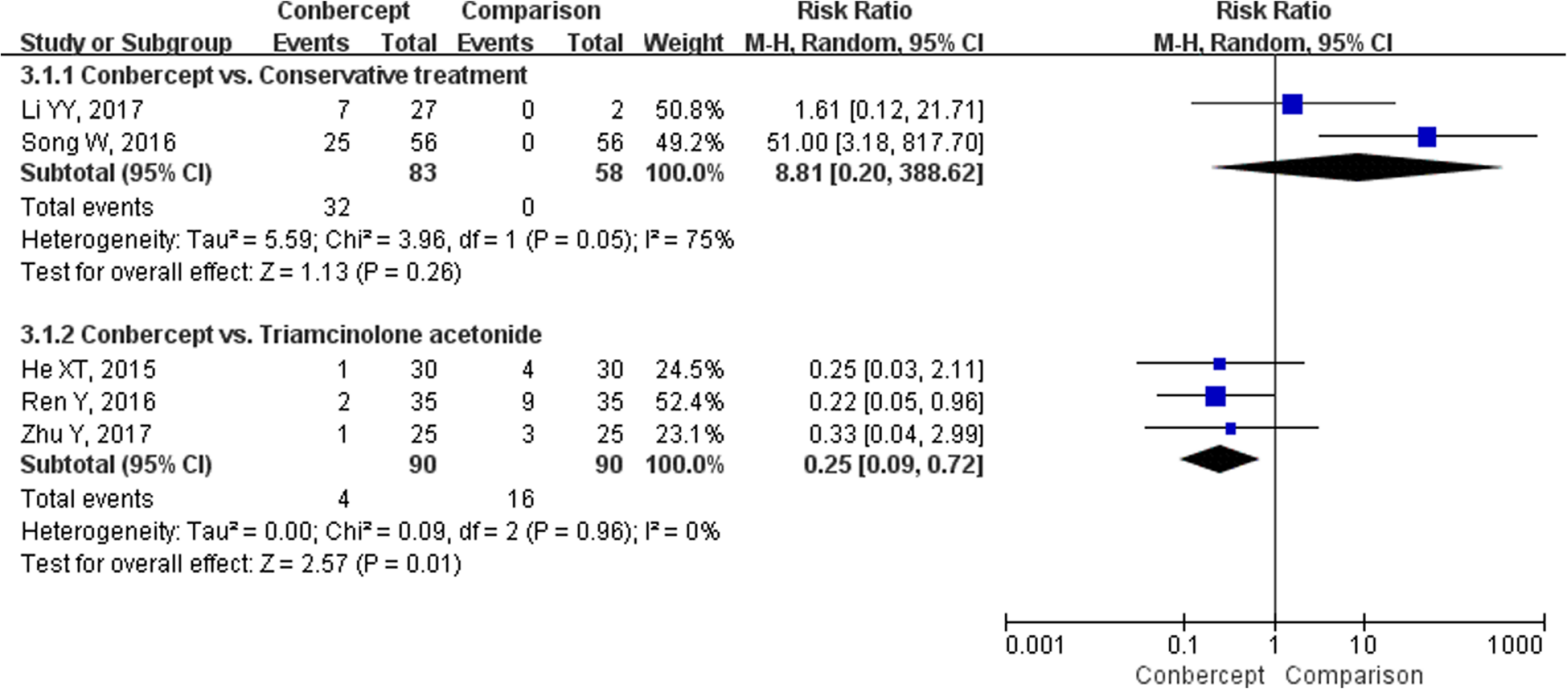
The forest plot of the incidence of adverse events

**Fig. 7:**
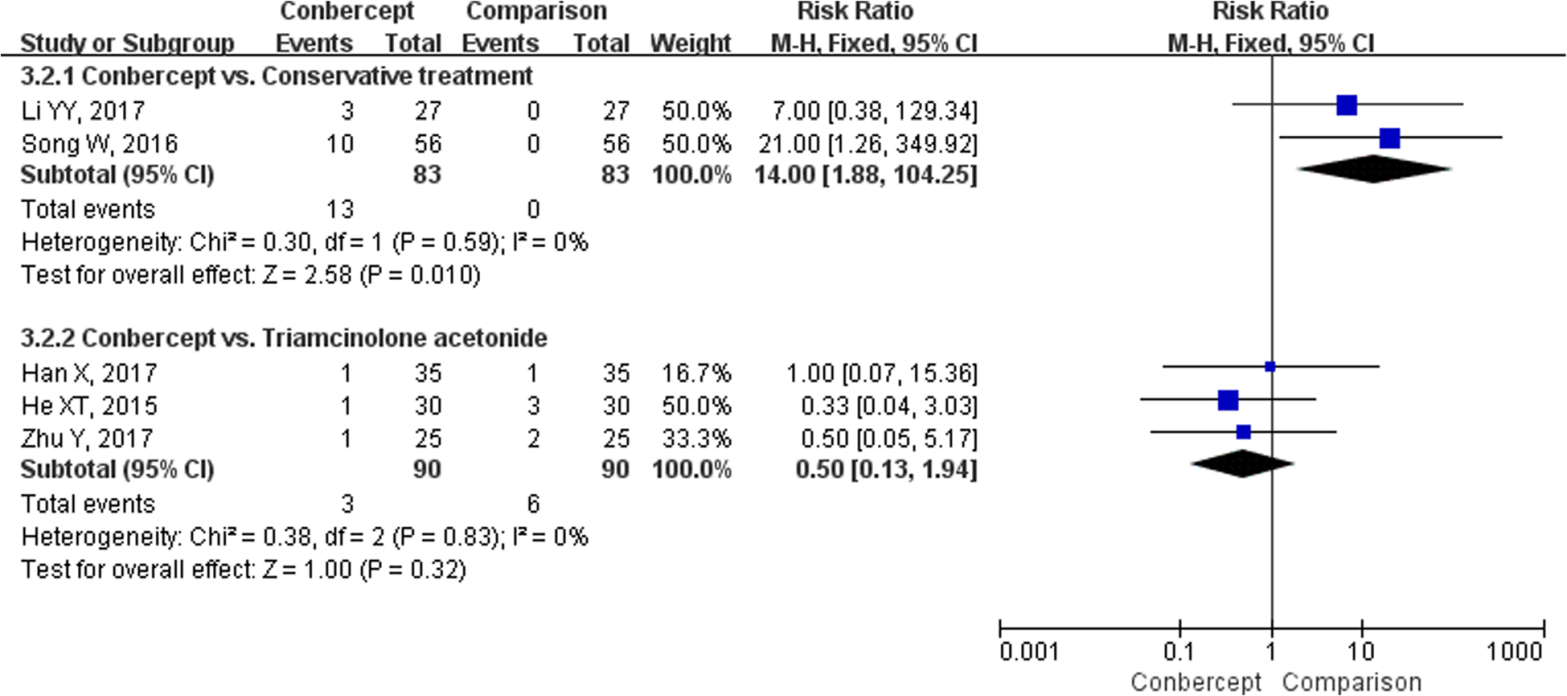
The forest plot of the incidence of increased intraocular pressure

**Fig. 8:**
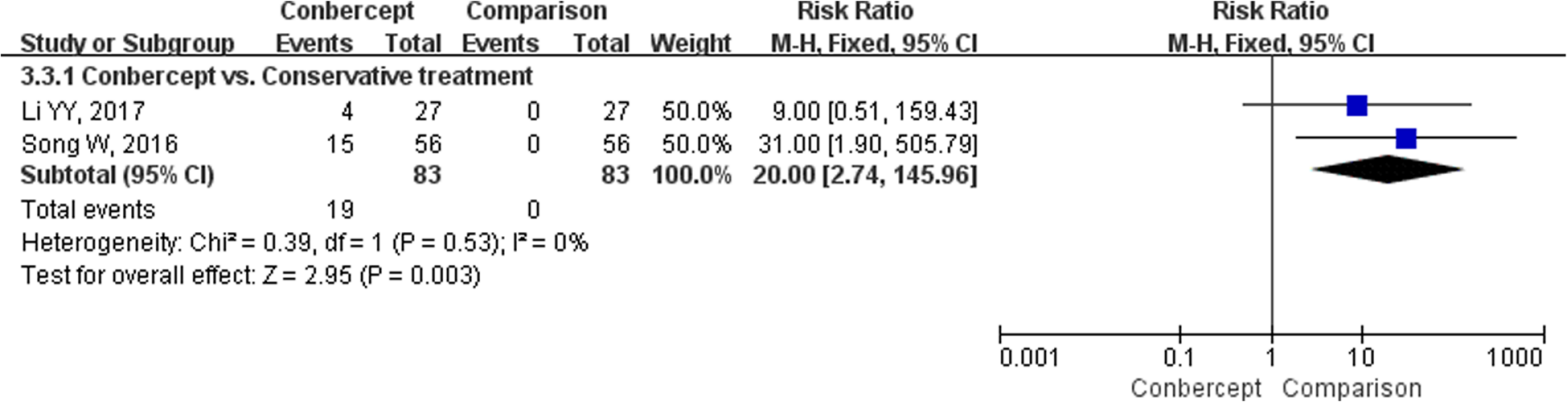
The forest plot of the incidence of ophthalmecchymosis

Eight studies [17, 19, 21, 24, 28, 30–32] (566 participants) reported the incidence of any AEs. Due to the significant heterogeneity in conbercept vs. conservative treatment subgroup (*I*^*2*^ = 75%, *P* = 0.05), the RRs were pooled with a random-effects model (Fig. 6). The incidence of AEs in the conbercept group was similar to the conservative treatment [*RR* = 8.81, 95% *CI* (0.20, 388.62)], ranibizumab [*RR* = 1.25, 95% *CI* (0.38, 4.12)] [21], and transpupillary thermotherapy groups [*RR* = 5.00, 95% *CI* (0.25, 101.81)] [28], but significantly lower than triamcinolone acetonide group [*RR* = 0.25, 95% *CI* (0.09, 0.72)]. None of the studies reported serious AEs, and the most common AEs were increased IOP and ophthalmecchymosis. The incidences of increased IOP (Fig. 7) and ophthalmecchymosis (Fig. 8) in the conbercept group were similar to ranibizumab [*RR* = 0.20, 95% *CI* (0.01, 3.97); *RR* = 1.50, 95% *CI* (0.27, 8.22), respectively] [21], transpupillary thermotherapy [*RR* = 3.00, 95% *CI* (0.13, 71.65); *RR* = 3.00, 95% *CI* (0.13, 71.65), respectively] [28], and triamcinolone acetonide [*RR* = 0.50, 95% *CI* (0.13, 1.94); *RR* = 3.00, 95% *CI* (0.13, 71.22) [32], respectively] groups, but were significantly higher than conservative treatment group [*RR* = 14.00, 95% *CI* (1.88, 104.25); *RR* = 20.00, 95% *CI* (2.74, 145.96), respectively].

### Sensitivity analysis

The sensitivity analyses were performed by pooling methods and statistical models regarding to test heterogeneity, and the results (BCVA, CRT, Plasma level of VEGF, and AEs) were robust.

## Discussion

This systematic review summarized the evidence of efficacy and safety of conbercept in patients with wet AMD. Our study suggests that the use of conbercept improves the BCVA compared to triamcinolone acetonide, and reduce the CRT compared to the other four therapies (conservative treatment, ranibizumab, transpupillary thermotherapy, and triamcinolone acetonide). The safety profile of conbercept is superior to triamcinolone acetonide, but similar to other controls. As to the anti-VEGF agents, conbercept seems to be more effective than ranibizumab in lowering the plasma level of VEGF.

Although the doses of conbercept reported in the RCTs ranged from 0.5 to 1.5 mg, a double-blinded, multicenter, controlled-dose RCT concluded that the mean improvement in BCVA, the mean reduction in CRT, and the incidences of AEs were of no significant difference between 0.5 and 2.0 mg conbercept dosing groups in treating neovascular AMD patients [35]. Accordingly, we supposed that different doses of conbercept did not cause the clinical heterogeneity.

The current evidence demonstrates the advantages of conbercept over the non-anti-VEGF agent controls; however, these controls were rarely used for treatment of wet AMD due to the relatively high recurrence rate. This systematic review indicated comparable efficacy in improving BCVA between conbercept and ranibizumab, which was consistent with a retrospective case-controlled study including 180 patients [36]. There were also many studies [20, 22, 27, 33, 34, 37] reporting naked vision as an outcome rather than BCVA. Naked vision was too susceptible to many other factors to be used as the outcome for wet AMD. Therefore we recommend BCVA should be set as the uniform outcome for measuring visual acuity in the future studies.

Our study also found that, compared with ranibizumab, conbercept significantly reduced the CRT, which was slightly inconsistent with Cui et al. [36]. The stronger effect of conbercept on reducing CRT might be on account of different mechanisms of action of the two anti-VEGF agents: ranibizumab, a fully humanized monoclonal antibody fragment, functions by blocking the receptor binding domains of all VEGF-A isoforms [38]; while conbercept, a novel recombinant fusion protein, binds to not only VEGF-A but also VEGF-B and PIGF [35]. Cui et al [36] also found a slightly more CRT improvement in the conbercept group than that in the ranibizumab group, but the difference was not statistically significant, which might be attributed to the influence of confounding factors and small sample size.

A prospective, interventional case series [39] including 28 patients concluded that conbercept significantly decreased serum VEGF level at 1 day and 1 week after injection, while ranibizumab had no significant effect on serum VEGF concentration, which was consistent with our study. The reduction in serum VEGF may affect conbercept’s systemic safety profile, but the result of meta-analysis did not show any significant difference between conbercept and ranibizumab, which was consistent with Cui et al [36]. Due to the small sample size and short follow-up period of included RCT, the safety of conbercept needs to be further evaluated by long-term, larger sample size study.

On the other hand, a cost-effectiveness analysis [40] based on a Markov model concluded that conbercept was a cost-effective alternative for the treatment of wet AMD in China, compared with ranibizumab. Considering the limitation of model and paucity of studies about life quality of patients with wet AMD, the pharmacoeconomic research in real-world population should be conducted in the future.

The limitations of this study must be acknowledged as follow: 1) Included RCT were all conducted in Chinese population because conbercept is only approved in China, which limited the representativeness of sample and generalization of the conclusions. Hence the efficacy and safety of conbercept needed to be evaluated in other racial population. 2) Included RCTs with small sample size and short-term follow-up phase were not sensitive enough to find rare AEs, so the safety of conbercept should be further-assessed in larger samples and longer follow-up. 3) The methodological quality of the primary studies was poor, especially without descriptions about allocation concealment and blinding methods as well as registration information. In addition, the overall small size of all studies contributing to any one treatment effect limited the power of statistical tests in meta-analysis. Therefore, prospective, multicenter, RCTs with larger samples and better methodological design are urgently needed in this therapeutic area.

## Conclusion

In conclusion, current evidence suggests that conbercept is a promising option for the treatment of wet AMD. Due to the limitations of included studies, further studies (RCTs with larger sample and better methodological design) are warranted to compare the efficacy, long-term safety and cost-effectiveness between conbercept and other anti-VEGF agents (e.g. ranibizumab) in different populations. And researchers should increase focus on patient-reported outcomes (eg. quality of life) in the further research.

## Additional file

Additional file 1: **Table S1.** PRISMA 2009 Checklist. **Table S2.** Searching Strategy. (DOC 65 kb)

## Abbreviations

AAO: American Academy of Ophthalmology
AEs: Adverse events
AMD: Age-related macular degeneration
BCVA: Best-corrected visual acuity
CENTRAL: Cochrane Central Register of Controlled Trials
CIs: Confidence intervals
CNKI: China National Knowledge Infrastructure
CRT: Central retinal thickness
EURETINA: European Society of Retina Specialists
MDs: Mean differences
OCT: Optical coherence tomography
PCV: Polypoidal choroidal vasculopathy
PDT: Photodynamic therapy
PIGF: Placental growth factor
PRISMA: Preferred Reporting Items for Systematic reviews and Meta-Analyses
RCTs: Randomized controlled trials
RRs: Risk ratios
VEGF: Vascular endothelial growth factor
